# Improving the quality of predictive models in small data GSDOT: A new algorithm for generating synthetic data

**DOI:** 10.1371/journal.pone.0265626

**Published:** 2022-04-07

**Authors:** Georgios Douzas, Maria Lechleitner, Fernando Bacao

**Affiliations:** NOVA Information Management School, Campus de Campolide, Lisboa, Portugal; Victoria University of Wellington, NEW ZEALAND

## Abstract

In the age of the data deluge there are still many domains and applications restricted to the use of small datasets. The ability to harness these small datasets to solve problems through the use of supervised learning methods can have a significant impact in many important areas. The insufficient size of training data usually results in unsatisfactory performance of machine learning algorithms. The current research work aims to contribute to mitigate the small data problem through the creation of artificial instances, which are added to the training process. The proposed algorithm, Geometric Small Data Oversampling Technique, uses geometric regions around existing samples to generate new high quality instances. Experimental results show a significant improvement in accuracy when compared with the use of the initial small dataset as well as other popular artificial data generation techniques.

## 1 Introduction

Insufficient size of datasets is a common issue in many supervised learning tasks [1, 2]. The limited availability of training samples can be caused by different factors. First, data is becoming an increasingly expensive resource [3] as the process to retain them is getting more complex due to strict privacy regulations such as the General Data Protection Regulation (GDPR) [4]. Additionally, the small dataset problem can be found in numerous industries where organizations simply do not have access to a reasonable amount of data. For example manufacturing industries are usually dealing with a small number of samples in the early stages of product development while health care organizations have to work with different kinds of rare diseases, where very few records are available [2].

In machine learning, researchers are usually concerned with the design of sophisticated learning algorithms when aiming to improve prediction performance. However, increasing the sample size is often a more effective approach. A rule of thumb is that “a dumb algorithm with lots and lots of data beats a clever one with modest amounts of it” [5]. Generally, a small number of training samples is characterized by a loose data structure with multiple information gaps. This lack of information negatively impacts the performance of machine learning algorithms [6]. Consequently, the knowledge gained from models trained with small sample sizes is considered unreliable as well as imprecise and does not lead to a robust performance [2].

Considering the size of data, there are two types of problems: The first, is the insufficiency of data belonging to one or more of the classes (imbalance learning problem) for a binary or multi-class classification task while the second is the small size of the whole dataset (small data problem) for any classification or regression task [7]. In both cases, the performance of machine learning models is affected [8]. In this work, we consider only the second type of problems i.e. the small data problem proposing an efficient algorithm, GSDOT, that increases the classification performance.

A theoretical definition of “small” can be found in statistical learning theory by Vapnik. A sample size is defined as small, if the ratio between the number of training samples and Vapnik-Chervonenkis (VC) dimensions is approximately less than 20. VC dimensions are determined as the maximum number of vectors that can be separated into two classes in all possible ways by a set of functions [9].

**Fig 1:**
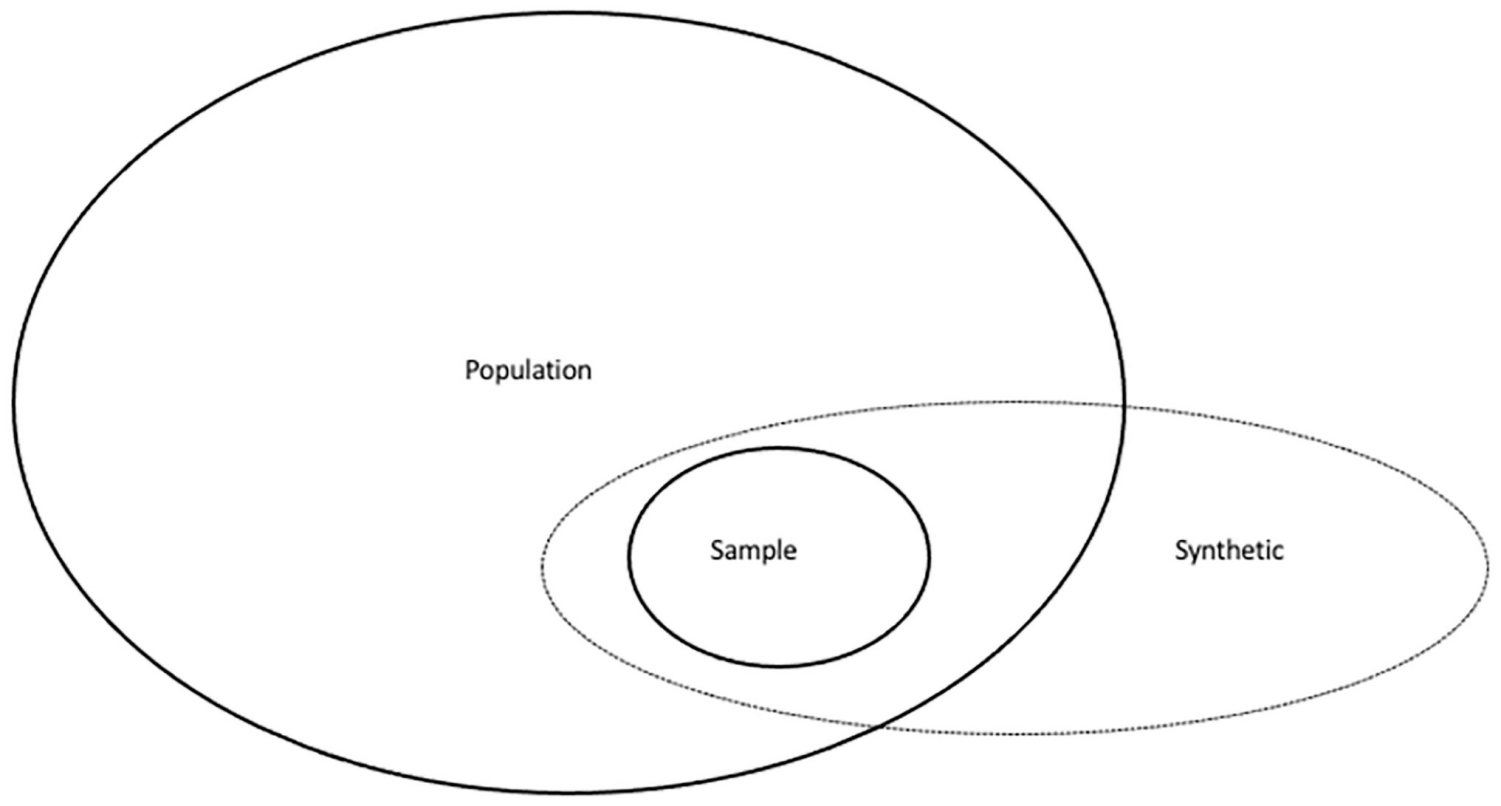
Relationship between population, sample and synthetic data [10].

Under-representation of observations in the sample set can be solved in different ways. Techniques to artificially add information by extending the sample size, and eventually improving the performance of the algorithms, can translate into significant improvements in many application domains [7]. However, it is important to note that the challenge in artificial data generation is to create data which extend the training set without creating noise [10]. Additionally, generating artificial data will only work if the initial sample is representative of the underlying population. Fig 1 shows the relationship between population, sample and synthetic data.

The next sections will describe an effective way to tackle the small data problem. Specifically, the focus in this paper is the case of binary classification tasks with the objective to generate artificial data for both of the classes, called arbitrarily the positive and negative class. The application for the multi-class case is also straightforward and it is based on the binarization of the problem through the one-vs-all approach. On the other hand, regression tasks require an extensive modification of the data generation process and they will be a topic of future research.

In section 2, the previously studied solutions are reviewed, while a detailed description of the proposed method is presented in section 3. This is followed by the research methodology and the experimental results in sections 4 and 5. Finally, the conclusions of the paper are presented in section 6.

## 2 Related work

Several methods to increase the data size have been presented by the research community. In this section, the most important approaches to deal with the small data problem are presented. We start by describing fuzzy theories, which have historically been the most used approach. Next, we look at the resampling mechanism, which mainly consists of bootstrapping techniques, and finally, we review oversampling methods that can be a valuable option to increase the sample size in small datasets.

### 2.1 Fuzzy theory

Many artificial sample generation techniques presented in the literature are based on fuzzy theory [2]. The fuzzy set theory defines a strict mathematical framework to generalize the classical notion of a dataset providing a wide scope of applicability, especially in the fields of information processing and pattern classification [11]. Based on this concept, several methods have emerged in the last decade to estimate or approximate functions which are generating artificial samples for small datasets.

The fundamental concept of creating synthetic data is called Virtual Sample Generation (VSG) and was originally proposed by [1]. The introduction of virtual examples expands the effective training set size and can therefore help to mitigate the learning problem. [1] showed that the process of creating artificial samples is mathematically equivalent to incorporating prior knowledge. The concept was applied on object recognition by transforming the views of 3D-objects and therefore generating artificial samples.

Based on the above approach, several closely related studies were developed for manufacturing environments. The first method to overcome scheduling problems, due to the lack of data in early stages of manufacturing systems, was the creation of a Functional Virtual Population (FVP) [12]. A number of synthetic samples was created, within a newly defined domain range. Although, the process was manually configured, its application dramatically improved the classification accuracy of a neural network.

[13] proposed the Diffusion-Neural-Network (DNN) method, an approach that fuzzifies information in order to extend a small dataset. It combines the principle of information diffusion by [14] with traditional Neural Networks to approximate functions. The information diffusion method partially fills the information gaps by using fuzzy theory to represent the similarities between samples and subsequently derive new ones.

In order to fully fill the information gaps, Mega-Trend-Diffusion (MTD) [3] combines data trend estimation with a diffusion technique to estimate the domain range, thus avoiding overestimation. It diffuses a set of data instead of each sample individually. It is considered as an improvement of DNN and was initially developed to improve early flexible manufacturing system scheduling accuracy. In further research, MTD was widely used as a synthetic sample generation method and was recognized as an effective way to deal with small datasets [2].

A drawback of MTD is that only considers the data attributes as independent and does not deal with their relationships. Genetic Algorithm Based Virtual Sample Generation was proposed that takes the relationship among the attributes into account and explores the integrated effects of attributes instead of dealing with them individually. The algorithm has three steps: Initially, samples are randomly selected to determine the range of each attribute by using MTD functions. Next, a Genetic Algorithm is applied to find the most feasible virtual samples. Finally, the average error of these new samples is calculated. The results outperformed the ones using MTD and also showed better performance in prediction than in the case of no generation of synthetic samples [15, 16].

### 2.2 Bootstrapping Procedure or Random OverSampling

An alternative approach to fuzzy theory as well the most well-known artificial sample generation method is the Bootstrapping Procedure [2] or Random OverSampling (ROS). The main difference to the previously presented techniques is that ROS expands the training set by duplicating instances from the original dataset [17]. The selection is done with replacement, thus it allows the algorithms to use the same sample more than one time. However, ROS may cause overfitting when applied to small data because it repetitively uses the same information [18, 19]. Nevertheless, [20] applied ROS in batch process industries where it was shown that it may help mitigate the small data problem.

## 3 Proposed method

Compared to the previous section, a different approach to fill information gaps is the creation of new instances and not copies of the existing ones like in ROS. These methods were originally developed in the context of machine learning to deal with the imbalanced learning problem. Therefore, their origin comes from a different research community than the fuzzy and bootstrapping methods presented above.

In this section, we present Geometric Small Data Oversampling Technique (GSDOT) as a novel data generation procedure suitable for the small data problem. The data generation mechanism of GSDOT is based on the oversampling algorithm Geometric SMOTE (G-SMOTE) [21]. GSDOT is applied on the entire dataset, independent from the class distribution. Therefore, GSDOT constitutes a new algorithm that generates artificial data for all the classes in the dataset.

GSDOT algorithm randomly generates artificial data within a geometric region of the input space. The size of this area is derived from the distance of the selected sample, either from the positive or negative class, to one of its nearest neighbors, whereas the shape is determined by the hyperparameters called *truncation factor* and *deformation factor*. Additionally, the *selection strategy* hyperparameter modifies the selection process and also affects the size of the geometric region. Details of hte algorithm are provided below.

### 3.1 GSDOT algorithm

The inputs of the GSDOT algorithm are sets of the positive and negative class samples *S*_*pos*_, *S*_*neg*_ respectively, the three geometric hyper-parameters *truncation factor*, *deformation factor* and *selection strategy* as well as the number of generated samples for the positive class *N*_*pos*_ and for the negative class *N*_*neg*_. A sensible choice for the last two inputs, used also in the experimental procedure below, is to preserve the class distribution in the resampled dataset. The GSDOT algorithm can be generally described in the following steps:

1. An empty set *S*_*gen*_ is initialized. *S*_*gen*_ will be populated with artificial data from both classes.
2. *S*_*pos*_ is shuffled and the process described below is repeated *N*_*pos*_ times until *N*_*pos*_ artificial points have been generated.
  2.1. A positive class instance **x**_*center*_ is selected randomly from *S*_*pos*_ as the center of the geometric region.
  2.2. Depending on the values of *α*_*sel*_ (*positive*, *negative* or *combined*), this step results in a randomly selected sample **x**_*surface*_ which belongs to either *S*_*pos*_ or *S*_*neg*_.
  2.3. A random point **x**_*gen*_ is generated inside the hyperspheroid centered at **x**_*center*_. The major axis of the hyper-spheroid is defined by **x**_*surface*_ − **x**_*center*_ while the permissible data generation area as well as the rest of geometric characteristics are determined by the hyperparameters *truncation factor* and *deformation factor*.
  2.4. **x**_*gen*_ is added to the set of generated samples **S**_*gen*_.
3. Step 2 is repeated using the substitution *pos* ↔ *neg* until *N*_*neg*_ artificial points have been generated.

### 3.2 Considerations

**Fig 2:**
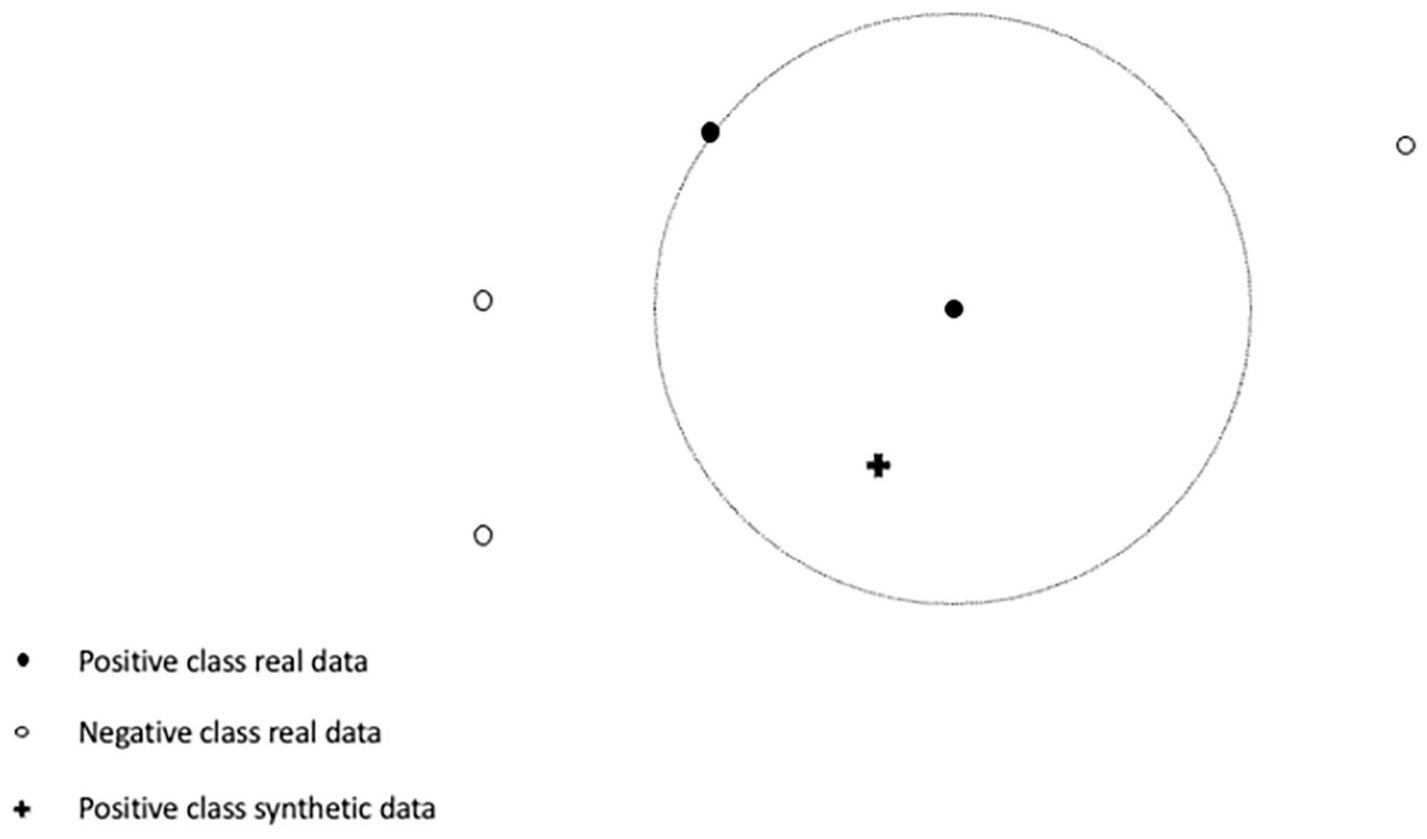
The GSDOT data generation mechanism when positive class samples are generated. The process is repeated for the negative class.

As it is shown above, GSDOT algorithm applies independently the G-SMOTE data generation process for both the positive and negative classes. The above description of step 2, that constitutes the data generation mechanism, excludes mathematical formulas and details which can be found in [21]. Fig 2 shows an example of the GSDOT data generation process when positive class data generation is considered.

## 4 Research methodology

The main objective of this work is to compare GSDOT to other algorithms that deal with the the small data problem. Therefore, we use a variety of datasets, metrics and classifiers to evaluate the performance of the various methods. A description of this set-up, the experimental procedure as well as the software implementation is provided in this section.

### 4.1 Experimental data

The ten datasets used to test the performance of GSDOT are retrieved from UCI Machine Learning Repository [22]. The focus on their selection lies on binary classification problems with a balanced distribution of the two classes. In order to assure generalizability of the results, the datasets are related to different topics such as health care, finance, business and physics. Details of the datasets are presented in Table 1.

**Table 1:** Description of the datasets.

Dataset	Number of samples	Number of attributes	Area
Arcene	900	10.000	Health Care
Audit	776	18	Business
Banknote Authentication	1.372	5	Finance
Spambase	4.610	57	Business
Breast Cancer	699	10	Health Care
Indian Liver Patient	583	10	Health Care
Ionosphere	351	34	Physics
MAGIC Gamma Telescope	19.020	11	Physics
Musk	6.598	168	Physics
Parkinsons	197	23	Health Care

The approach to test whether oversamplers, and particularly GSDOT, are able to produce high quality artificial data, is to generate randomly undersampled versions of the above datasets and try to reconstruct them. Specifically, random sampling of 50%, 75%, 90% and 95% is applied on them, called undersampling ratio, followed by their enhancement with artificial data that are created from the various oversampling methods. The details of the process are presented in subsection 4.4.

### 4.2 Evaluation metrics

To evaluate the performance of GSDOT, the experiment includes two different metrics. The first choice is *Accuracy* as it is one of the most common metrics for the evaluation of classification models [23]. *Accuracy* measures the ratio of correct predictions over the total number of instances. The mathematical formula is the following:

$$\text{Accuracy}=\frac{TP+TN}{TP+TN+FP+FN}$$

where *TP*, *TN*, *FP*, *FN* denote the number of correctly classified positive, negative and misclassified negative, positive instances, respectively. *Accuracy* might be inappropriate for datasets with a significant difference between the number of positive and negative classes since rare classes have a small impact to the final outcome compared to the majority classes. To make sure the contribution in the accuracies of the two classes stay relatively balanced, we include the geometric mean score (*G-Mean*) as a second measure. *G-Mean* is the geometric mean of *sensitivity* and *specificity*:

$$\text{G-Mean}=\sqrt{sensitivity\times specificity}=\sqrt{\frac{TP}{TP+FN}\times \frac{TN}{TN+FP}}$$

### 4.3 Machine learning algorithms

For the evaluation of the oversampling methods, a variety of classifiers are included to ensure that the results are independent of their characteristics. Specifically, the experiment is conducted using the following four classifiers: Logistic Regression (LR) [24], K-Nearest Neighbors (KNN) [25], Decision Tree (DT) [26] and Gradient Boosting (GB) [27].

To deal with the small data problem, GSDOT is compared to three other algorithms. One of them, ROS is chosen for its simplicity. As explained in the sections above, although GSDOT is a novel algorithm, its data generation mechanism is based on G-SMOTE. Besides G-SMOTE, there are several other informed oversampling algorithms presented in the literature. The first method to be proposed and still the most popular is the Synthetic Minority Oversampling TEchnique (SMOTE) [28]. Numerous variants of SMOTE have been created, increasing its status [29], with one of the most popular and effective variants being Borderline SMOTE (B-SMOTE) [30]. In the case of the small data problem, when SMOTE and B-SMOTE are used, the data generation process is trivially extended to include not only the minority classes but also the majority class [19]. We include both of them in the experimental procedure. Finally, the benchmark results (B-MARK) of using the original data are also included, as well as the case when no synthetic data are generated and the classifiers are trained using the undersampled data (NONE).

### 4.4 Experimental procedure

As explained above, the main goal of the paper is to evaluate how well GSDOT algorithm, as presented in subsection 3.1, compares to other methods, when small datasets are enhanced with artificial samples.

The performance of the classifiers is assessed using *k*-fold cross-validation scores with *k* = 5. Each dataset *D* is randomly splitted into *k* subsets (folds) *D*_1_, *D*_2_, ⋯, *D*_*k*_ of approximately equal size. Each fold is used as a test set and the remaining folds are used to train the model. The process is repeated in *k* stages, until each *D*_*k*_ is used as a validation set [31]. The experimental procedure for an arbritary dataset and cross-validation stage is described below:

1. The *k* − 1 folds are undersampled using an undersampling ratio of 50%, 75%, 90% and 95%, equal to the percentage of the dataset that is removed (1). Alternatively, no undersampling is applied and the original data are presented to the classifiers, a case identified as B-MARK (2).
2. Synthetic data generation is applied to the undersampled data (3) of the previous step that increases their size and class distribution back to the initial (4). Alternatively, no synthetic data are generated and the small data are presented to the classifiers, a case identified as NONE (5).
3. The resampled data of the previous step as well as the data from two special cases as described above are used to train the classifiers.
4. The classifiers are evaluated on the remaining fold of step 1.

**Fig 3:**
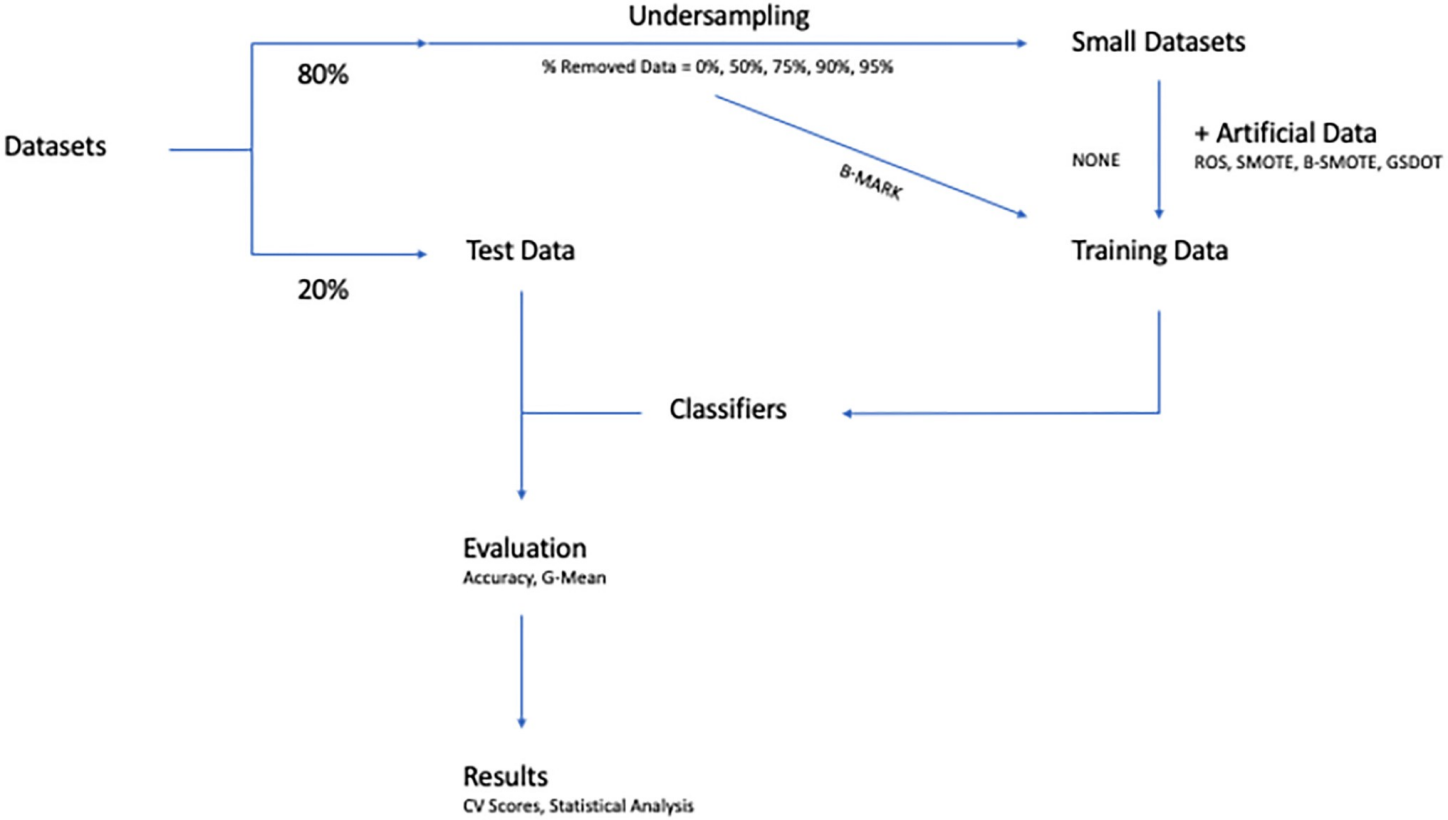
Visualization of the experimental procedure.

Fig 3 provides a schematic represenation of the experimental procedure:

This procedure results in a cross validation score for each combination of dataset, classifier, synthetic data generation method and evaluation metric. It is also repeated three times and the average cross-validation score is calculated across the three runs. The initialization in each of the runs is random, including the undersampling step of the process and all random parameters of the machine learning algorithms. The algorithms used in the experiment have various hyperparameters that yield different scores. The maximum of these scores is reported.

In order to confirm the statistical significance of the experimental results, the Friedman test as well as the Holm test [32] are applied. Ranking scores are assigned to each synthetic data generation method, as well as the B-MARK and NONE cases, with scores of 1 to 5 for the best and worst performing methods, respectively. The Friedman test is a non-parametric procedure that compares the average rankings of the algorithms under the null hypothesis that all show identical performance independent of the selected classifier and evaluation metric. If the null-hypothesis is rejected to our favor, we proceed with the Holm test. The Holm test acts as a post-hoc test for the Friedman test for controlling the family-wise error rate when all algorithms are compared to a control method. It is a powerful non-parametric test in situations where we want to test whether a newly proposed method is better than existing ones. The control method in our case is the proposed GSDOT method and is tested under the null hypothesis that it performs similarly to the rest of synthetic data generation methods for every combination of classifier and metric.

### 4.5 Software implementation

The implementation of the experimental procedure was based on the Python programming language, using the Scikit-Learn [33] and Imbalanced-Learn [34] libraries. All functions, algorithms, experiments and results reported are provided at the GitHub repository of the project. Additionally, the Research-Learn library provides a framework to implement comparative experiments, also being fully integrated with the Scikit-Learn ecosystem.

## 5 Results and discussion

In this section the performance of the different oversamplers and the results of the statistical tests are presented and analyzed.

### 5.1 Comparative presentation

The mean cross validation scores and the standard error across all datasets per classifier, metric and undersampling ratio (Ratio) are presented in Table 2. The Ratio is included in order to evaluate how the methods perform as the dataset size diminishes. As explained above, we also include the B-MARK method that represents the performance of the classfiers on the original dataset. The B-MARK method is expected to obtain the best results by design. Therefore, the highest scores for each row, excluding the B-MARK scores, are highlighted.

**Table 2:** Results for mean cross validation scores of all methods.

Ratio	Classifier	Metric	NONE	ROS	SMOTE	B-SMOTE	GSDOT	B-MARK
50	LR	ACCURACY	0.91 ± 0.03	0.91 ± 0.03	0.91 ± 0.02	0.91 ± 0.03	**0.92** ± 0.02	0.92 ± 0.02
50	LR	G-MEAN	0.88 ± 0.04	0.88 ± 0.04	**0.89** ± 0.04	**0.89** ± 0.04	**0.89** ± 0.04	0.90 ± 0.04
50	KNN	ACCURACY	0.88 ± 0.03	0.88 ± 0.03	**0.89** ± 0.03	0.88 ± 0.03	**0.89** ± 0.03	0.90 ± 0.03
50	KNN	G-MEAN	0.84 ± 0.04	0.85 ± 0.04	**0.86** ± 0.04	0.85 ± 0.04	**0.86** ± 0.04	0.87 ± 0.04
50	DT	ACCURACY	0.88 ± 0.04	0.88 ± 0.04	0.88 ± 0.04	0.88 ± 0.04	**0.90** ± 0.03	0.90 ± 0.03
50	DT	G-MEAN	0.86 ± 0.05	0.86 ± 0.05	0.87 ± 0.05	0.87 ± 0.05	**0.89** ± 0.04	0.89 ± 0.03
50	GBC	ACCURACY	0.91 ± 0.04	0.92 ± 0.03	0.92 ± 0.03	0.91 ± 0.04	**0.93** ± 0.03	0.94 ± 0.02
50	GBC	G-MEAN	0.90 ± 0.04	0.90 ± 0.04	0.91 ± 0.03	0.90 ± 0.04	**0.92** ± 0.03	0.93 ± 0.03
75	LR	ACCURACY	**0.90** ± 0.03	0.89 ± 0.03	0.89 ± 0.03	0.89 ± 0.03	**0.90** ± 0.03	0.92 ± 0.02
75	LR	G-MEAN	0.86 ± 0.05	0.86 ± 0.05	**0.87** ± 0.04	**0.87** ± 0.04	**0.87** ± 0.04	0.90 ± 0.04
75	KNN	ACCURACY	0.86 ± 0.04	0.86 ± 0.04	**0.87** ± 0.04	0.85 ± 0.04	**0.87** ± 0.04	0.90 ± 0.03
75	KNN	G-MEAN	0.80 ± 0.06	0.82 ± 0.05	**0.84** ± 0.04	0.83 ± 0.05	**0.84** ± 0.04	0.87 ± 0.04
75	DT	ACCURACY	0.86 ± 0.05	0.86 ± 0.05	0.86 ± 0.05	0.85 ± 0.06	**0.89** ± 0.04	0.90 ± 0.03
75	DT	G-MEAN	0.83 ± 0.06	0.84 ± 0.05	0.84 ± 0.06	0.83 ± 0.06	**0.86** ± 0.05	0.89 ± 0.03
75	GBC	ACCURACY	0.87 ± 0.05	0.88 ± 0.05	0.88 ± 0.05	0.88 ± 0.05	**0.90** ± 0.04	0.94 ± 0.02
75	GBC	G-MEAN	0.85 ± 0.06	0.85 ± 0.06	0.86 ± 0.05	0.85 ± 0.06	**0.89** ± 0.04	0.93 ± 0.03
90	LR	ACCURACY	0.86 ± 0.04	0.86 ± 0.04	0.86 ± 0.04	0.85 ± 0.04	**0.87** ± 0.04	0.92 ± 0.02
90	LR	G-MEAN	0.81 ± 0.06	0.82 ± 0.06	0.82 ± 0.06	0.82 ± 0.05	**0.83** ± 0.06	0.90 ± 0.04
90	KNN	ACCURACY	0.81 ± 0.05	0.82 ± 0.05	0.82 ± 0.05	0.81 ± 0.05	**0.83** ± 0.05	0.90 ± 0.03
90	KNN	G-MEAN	0.69 ± 0.10	0.76 ± 0.07	**0.78** ± 0.06	0.74 ± 0.09	**0.78** ± 0.06	0.87 ± 0.04
90	DT	ACCURACY	0.84 ± 0.05	0.83 ± 0.05	0.83 ± 0.06	0.83 ± 0.05	**0.87** ± 0.04	0.90 ± 0.03
90	DT	G-MEAN	0.81 ± 0.06	0.81 ± 0.06	0.80 ± 0.06	0.80 ± 0.06	**0.84** ± 0.05	0.89 ± 0.03
90	GBC	ACCURACY	0.84 ± 0.06	0.84 ± 0.06	0.84 ± 0.06	0.84 ± 0.05	**0.88** ± 0.04	0.94 ± 0.02
90	GBC	G-MEAN	0.82 ± 0.06	0.81 ± 0.06	0.81 ± 0.07	0.81 ± 0.06	**0.86** ± 0.05	0.93 ± 0.03
95	LR	ACCURACY	0.83 ± 0.05	0.83 ± 0.05	0.83 ± 0.05	0.83 ± 0.04	**0.84** ± 0.05	0.92 ± 0.02
95	LR	G-MEAN	0.75 ± 0.08	0.76 ± 0.07	0.76 ± 0.07	**0.77** ± 0.07	0.76 ± 0.08	0.90 ± 0.04
95	KNN	ACCURACY	0.79 ± 0.05	0.79 ± 0.05	**0.81** ± 0.05	0.79 ± 0.05	**0.81** ± 0.05	0.90 ± 0.03
95	KNN	G-MEAN	0.60 ± 0.13	0.69 ± 0.09	0.71 ± 0.09	**0.74** ± 0.06	0.73 ± 0.07	0.87 ± 0.04
95	DT	ACCURACY	0.81 ± 0.05	0.81 ± 0.05	0.82 ± 0.05	0.81 ± 0.05	**0.85** ± 0.05	0.90 ± 0.03
95	DT	G-MEAN	0.77 ± 0.06	0.78 ± 0.06	0.78 ± 0.06	0.78 ± 0.06	**0.81** ± 0.06	0.89 ± 0.03
95	GBC	ACCURACY	0.82 ± 0.05	0.83 ± 0.05	0.83 ± 0.05	0.82 ± 0.05	**0.85** ± 0.05	0.94 ± 0.02
95	GBC	G-MEAN	0.77 ± 0.07	0.78 ± 0.07	0.78 ± 0.07	0.78 ± 0.07	**0.81** ± 0.07	0.93 ± 0.03

Table 2 shows that GSDOT outperforms all other methods, almost for all combinations of classifiers and metrics. Throughout the scores we can observe that all methods have a better performance as the dataset increase their size i.e. the Ratio gets smaller. Particularly, the scores of GSDOT are the closest to the ones of the B-MARK results, which implies that it is able to reconstruct the original dataset more effectively compared to the rest of the synthetic data generation methods.

Table 3 presents the mean and standard error of percentage difference between GSDOT and NONE. It shows that GSDOT performs significantly better compared to the case where no synthetic data generation is applied for every combination of undersampling ratio, classifier and metric. Particularly, the performance gap increases for higher undersampling ratios.

**Table 3:** Results for percentage difference between GSDOT and NONE.

Ratio	Classifier	Metric	Difference
50	LR	ACCURACY	0.52 ± 0.27
50	LR	G-MEAN	0.36 ± 0.14
50	KNN	ACCURACY	1.30 ± 0.45
50	KNN	G-MEAN	2.48 ± 0.96
50	DT	ACCURACY	2.58 ± 1.02
50	DT	G-MEAN	3.72 ± 1.61
50	GBC	ACCURACY	2.75 ± 1.42
50	GBC	G-MEAN	2.90 ± 1.46
75	LR	ACCURACY	0.40 ± 0.15
75	LR	G-MEAN	1.05 ± 0.58
75	KNN	ACCURACY	1.93 ± 0.50
75	KNN	G-MEAN	7.27 ± 4.51
75	DT	ACCURACY	4.13 ± 1.88
75	DT	G-MEAN	4.67 ± 1.97
75	GBC	ACCURACY	4.39 ± 2.51
75	GBC	G-MEAN	5.67 ± 3.00
90	LR	ACCURACY	1.41 ± 0.52
90	LR	G-MEAN	3.26 ± 1.58
90	KNN	ACCURACY	2.95 ± 1.21
90	KNN	G-MEAN	33.43 ± 26.93
90	DT	ACCURACY	4.47 ± 1.46
90	DT	G-MEAN	4.32 ± 1.88
90	GBC	ACCURACY	5.17 ± 2.48
90	GBC	G-MEAN	5.64 ± 2.35
95	LR	ACCURACY	1.40 ± 0.63
95	LR	G-MEAN	1.23 ± 3.71
95	KNN	ACCURACY	2.94 ± 1.28
95	KNN	G-MEAN	23.66 ± 20.31
95	DT	ACCURACY	5.00 ± 2.04
95	DT	G-MEAN	5.18 ± 1.79
95	GBC	ACCURACY	4.11 ± 1.96
95	GBC	G-MEAN	5.25 ± 2.43

A ranking score in the range 1 to 5 is assigned to each oversampler as well as the two special case NONE and B-MARK. The mean ranking across the datasets of all methods is presented in Table 4:

**Table 4:** Results for mean rankings of all methods.

Ratio	Classifier	Metric	NONE	RANDOM	SMOTE	B-SMOTE	GSDOT	B-MARK
50	LR	ACCURACY	4.64	4.64	3.07	5.14	**1.71**	1.79
50	LR	G-MEAN	5.14	4.57	**2.57**	4.14	2.71	1.86
50	KNN	ACCURACY	4.36	5.43	3.0	4.14	**2.14**	1.93
50	KNN	G-MEAN	4.71	5.0	3.0	4.0	**2.43**	1.86
50	DT	ACCURACY	4.43	4.57	3.79	4.71	**1.71**	1.79
50	DT	G-MEAN	4.79	4.64	3.36	4.64	**1.86**	1.71
50	GBC	ACCURACY	5.29	4.21	4.0	4.36	**1.79**	1.36
50	GBC	G-MEAN	5.21	4.5	3.93	4.21	**1.86**	1.29
75	LR	ACCURACY	4.0	4.64	3.86	5.36	**2.14**	1.0
75	LR	G-MEAN	4.43	4.86	3.71	4.57	**2.29**	1.14
75	KNN	ACCURACY	4.86	4.57	2.79	5.0	**2.21**	1.57
75	KNN	G-MEAN	5.43	4.57	2.57	4.57	**2.29**	1.57
75	DT	ACCURACY	4.14	4.29	4.14	5.0	**2.14**	1.29
75	DT	G-MEAN	4.43	4.0	4.14	4.86	**2.43**	1.14
75	GBC	ACCURACY	4.71	4.0	3.86	4.86	**2.43**	1.14
75	GBC	G-MEAN	4.86	4.14	4.0	4.43	**2.43**	1.14
90	LR	ACCURACY	4.21	4.29	3.64	5.43	**2.43**	1.0
90	LR	G-MEAN	5.14	4.29	3.86	4.43	**2.29**	1.0
90	KNN	ACCURACY	5.0	4.36	3.0	5.07	**2.57**	1.0
90	KNN	G-MEAN	5.43	4.57	2.57	5.0	**2.43**	1.0
90	DT	ACCURACY	4.21	4.36	4.21	5.21	**2.0**	1.0
90	DT	G-MEAN	4.5	4.07	4.36	4.93	**2.14**	1.0
90	GBC	ACCURACY	4.64	4.14	3.93	5.0	**2.29**	1.0
90	GBC	G-MEAN	4.43	4.14	4.14	5.0	**2.29**	1.0
95	LR	ACCURACY	4.29	4.71	3.29	5.14	**2.57**	1.0
95	LR	G-MEAN	4.64	4.79	3.29	4.43	**2.86**	1.0
95	KNN	ACCURACY	5.14	4.71	**2.57**	4.86	2.71	1.0
95	KNN	G-MEAN	5.57	4.29	3.0	4.29	**2.86**	1.0
95	DT	ACCURACY	5.36	4.29	3.93	4.43	**2.0**	1.0
95	DT	G-MEAN	5.14	4.29	3.86	4.43	**2.29**	1.0
95	GBC	ACCURACY	4.43	4.36	3.71	5.29	**2.21**	1.0
95	GBC	G-MEAN	4.5	4.5	3.64	4.86	**2.5**	1.0

The highest rankings for each row, excluding the B-MARK case, are highlighted. Looking at the table, GSDOT is ranked on the top place when comparing with NONE, ROS, SMOTE and B-SMOTE.

### 5.2 Statistical analysis

To confirm the significance of the above presented results we apply the Friedman test as well as the Holm Test on the above results. The application of the Friedman test is presented in Table 5:

**Table 5:** Results for Friedman test.

Classifier	Metric	p-value	Significance
LR	ACCURACY	1.2e-11	True
LR	G-MEAN	6.9e-08	True
KNN	ACCURACY	2.7e-12	True
KNN	G-MEAN	3.5e-13	True
DT	ACCURACY	2.9e-12	True
DT	G-MEAN	6.7e-11	True
GBC	ACCURACY	4.9e-11	True
GBC	G-MEAN	1.7e-09	True

Therefore, the null hypothesis of the Friedman test is rejected at a significance level of a = 0.05, i.e. the synthetic data generation methods do not perform similarly in the mean rankings for any combination of classifier and evaluation metric.

The Holm method is applied to adjust the p- values of the paired difference test with GSDOT algorithm as the control method. The results are shown in Table 6:

**Table 6:** The p-values of the Holm’s test.

Classifier	Metric	NONE	ROS	SMOTE	B-SMOTE
LR	ACCURACY	2.9e-03	7.6e-05	2.9e-03	5.4e-05
LR	G-MEAN	2.1e-01	2.1e-01	1.0e-00	1.0e-00
KNN	ACCURACY	2.7e-05	7.8e-08	1.4e-01	1.8e-03
KNN	G-MEAN	1.1e-02	3.3e-03	2.9e-01	2.9e-01
DT	ACCURACY	1.5e-05	1.5e-05	4.8e-05	3.3e-05
DT	G-MEAN	1.3e-05	4.4e-05	4.4e-05	4.4e-05
GBC	ACCURACY	2.2e-03	2.9e-03	5.8e-03	1.8e-03
GBC	G-MEAN	1.8e-03	3.9e-03	7.3e-03	7.3e-03

At a significance level of a = 0.05 the null hypothesis of the Holm’s test is rejected for 25 out 32 combinations. This indicates that the proposed method outperforms all other methods in most cases.

## 6 Conclusions

Many domains and applications continue to be limited to the use of small datasets. The insufficient size of training data usually results in inferior performance of machine learning algorithms. This paper proposes an effective solution to mitigate the small data problem in classification tasks. As shown above, the GSDOT algorithm has the ability to generate high quality artificial samples and improve the prediction accuracy of the classifiers used in the experiments. This improvement relates to the algorithm’s capability of increasing the diversity of new instances while avoiding the generation of noisy samples. An important point is that GSDOT significantly improves classification performance compared to the case where only the small data are used, for every combination of undersampling ratio, classifier and metric as shown in Table 2. Specifically, the full experimental results show that there is not a single instance where using the small data outperformed GSDOT. Table 3 also shows that the performance gap increases for higher undersampling ratios. This is a clear indication that, when using a small dataset, it is safe and appropriate to apply the the GSDOT algorithm, in order to generate artificial samples and improve the performance of classifiers. Also GSDOT outperforms standard artificial data generation approaches such as ROS and SMOTE, being closer to the B-MARK scores than any of them. As presented in Table 2, in 30 out of 32 combinations of classifiers and metrics, GSDOT outperforms all other methods. Finally, the statistical analysis of the experiments, Tables 5 and 6, confirms the dominance of the proposed algorithm. The GSDOT implementation is available as an open source project, so that the research community and data science practitioners can make use of it to improve the performance of machine learning algorithms.
